# Recent Advances in Alkaline Exchange Membrane Water Electrolysis and Electrode Manufacturing

**DOI:** 10.3390/molecules26216326

**Published:** 2021-10-20

**Authors:** Ester López-Fernández, Celia Gómez Sacedón, Jorge Gil-Rostra, Francisco Yubero, Agustín R. González-Elipe, Antonio de Lucas-Consuegra

**Affiliations:** 1 Laboratory of Nanotechnology on Surfaces and Plasma, Institute of Materials Science of Seville (CSIC-University Sevilla), Av. Américo Vespucio 49, E-41092 Sevilla, Spain; jorge.gil@icmse.csic.es (J.G.-R.); yubero@icmse.csic.es (F.Y.); arge@icmse.csic.es (A.R.G.-E.); 2 Department of Chemical Engineering, School of Chemical Sciences and Technologies, University of Castilla-La Mancha, Avda. Camilo José Cela 12, E-13071 Ciudad Real, Spain; celia.gsacedon@uclm.es

**Keywords:** anion exchange membrane, water electrolysis, catalyst fabrication methods, non-noble electrodes, hydrogen production, green hydrogen

## Abstract

Water electrolysis to obtain hydrogen in combination with intermittent renewable energy resources is an emerging sustainable alternative to fossil fuels. Among the available electrolyzer technologies, anion exchange membrane water electrolysis (AEMWE) has been paid much attention because of its advantageous behavior compared to other more traditional approaches such as solid oxide electrolyzer cells, and alkaline or proton exchange membrane water electrolyzers. Recently, very promising results have been obtained in the AEMWE technology. This review paper is focused on recent advances in membrane electrode assembly components, paying particular attention to the preparation methods for catalyst coated on gas diffusion layers, which has not been previously reported in the literature for this type of electrolyzers. The most successful methodologies utilized for the preparation of catalysts, including co-precipitation, electrodeposition, sol–gel, hydrothermal, chemical vapor deposition, atomic layer deposition, ion beam sputtering, and magnetron sputtering deposition techniques, have been detailed. Besides a description of these procedures, in this review, we also present a critical appraisal of the efficiency of the water electrolysis carried out with cells fitted with electrodes prepared with these procedures. Based on this analysis, a critical comparison of cell performance is carried out, and future prospects and expected developments of the AEMWE are discussed.

## 1. Introduction

Hydrogen represents a suitable energy vector to guarantee engine operation, electricity production, and heat supplies in domestic, transport, and industrial sectors [1,2,3]. Currently, the global demand for hydrogen is 70 Mt_H2_ per year (International Energy Agency) from which most production relies on the steam-methane (or other hydrocarbons) reforming. This procedure is responsible for emitting a minimum of 7 kg of CO_2_ per kg of H_2,_ an unmanageable figure in the quest for an effective reduction of carbon dioxide emissions [4,5]. For this reason, alternative hydrogen production processes using environmentally friendly routes with no waste emissions to the atmosphere constitute an urgent and unavoidable requirement. 

**Figure 1:**
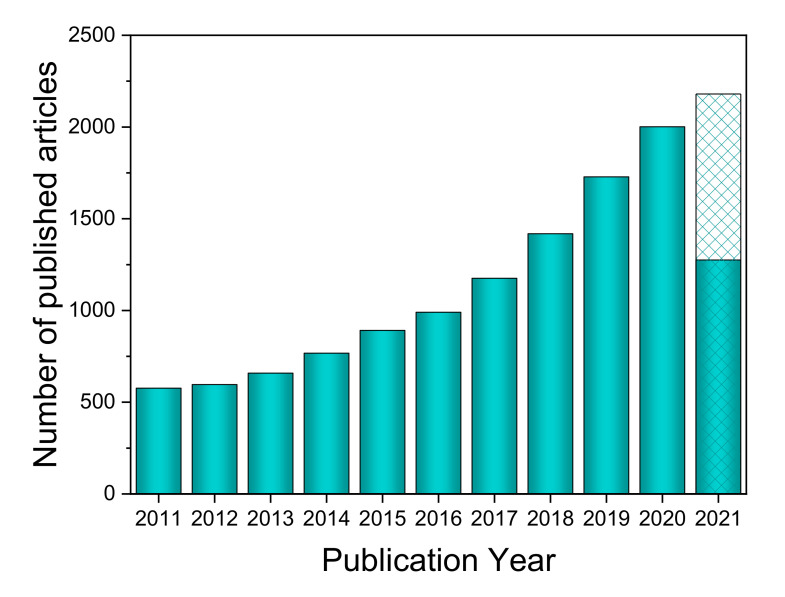
Trend of published articles on the topic of water electrolysis in the last 10 years (source Scopus 8 July 2021).

In this context, water electrolysis using intermittent renewable energy sources is emerging as a viable alternative, even at industrial scale, to the traditional methods for hydrogen production [6]. In recent years, the generalized interest in electrolysis has fostered a growing activity in the basic scientific aspects of the technology, as revealed by the increasing number of published papers in this topic (show Figure 1, from Scopus). 

Water electrolyzers can operate at high and low temperatures. Solid oxide electrolyzer cells (SOEC) operate at high temperatures, while three major technologies for water electrolyzers operate at low temperatures (around room temperature): alkaline water electrolysis (AWE), proton exchange membrane water electrolysis (PEMWE), and anion exchange membrane water electrolysis (AEMWE) [7,8]. Among other requirements, in this latter case, electrode catalysts must present high electrochemical activity and stability, be cheap, and have a secure supply. From an operational point of view, critical points to consider include a precise control over catalyst microstructure, composition, and chemical state. For practical exploitation, to reduce the cost related to the catalyst load in the electrodes, this must possess a high specific activity. These characteristics and requirements can be optimized by the use of appropriate preparation methods. In this review, we summarize the main concepts involved in the functioning of various low-temperature electrolyzers, paying special attention to the AEMWE and to the methods utilized so far for the preparation and processing of the electrode catalysts to enhance the cell performance. 

### 1.1. Low Temperature Water Electrolyzers

**Figure 2:**
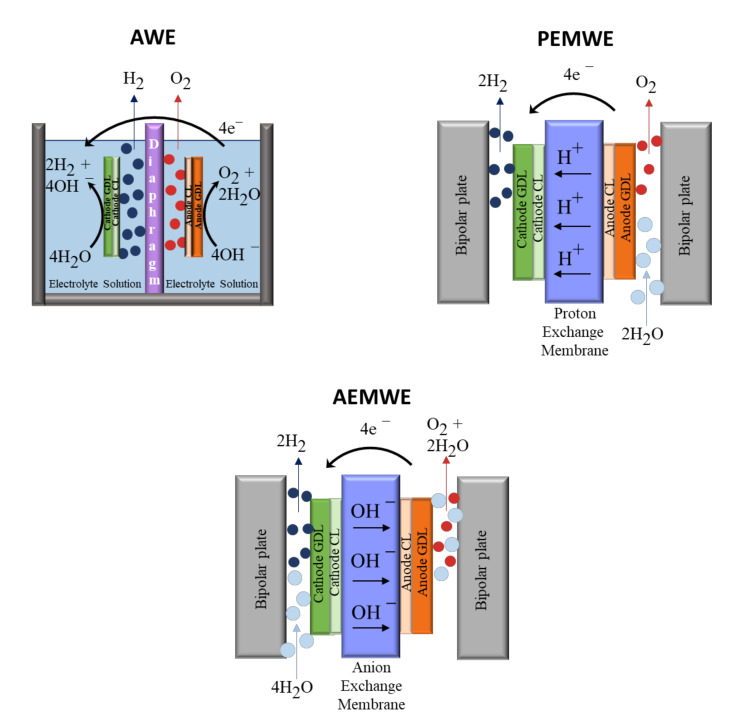
Schemes of typical cell configurations of low-temperature water electrolyzers.

Figure 2 shows a series of cell schemes utilized for various kinds of low-temperature water electrolysis. Their most relevant features are summarized below. 

#### 1.1.1. Alkaline Water Electrolysis (AWE)

Alkaline water electrolysis is the most traditional water electrolysis technology. It runs at low temperatures (60–80 °C), commonly with KOH at very high concentrations (20–40 % KOH) as a liquid electrolyte [6,9,10]. Cells for this process basically consist of two separate chambers. The cells incorporate a diaphragm, which is permeable to water and to hydroxide ions and serves to separate the anodic and cathodic chambers, where a suitable anode and cathode are located. Unfortunately, the diaphragms do not completely prevent the cross-over of the gases nor that some oxygen arrives to the cathode where it can combine with hydrogen to form water. This results in a decrease in the overall cell efficiency and may cause safety-related issues [6]. 

The AWE technology is compatible with non-noble metal catalyst electrodes [11]. Transition metal-based catalysts incorporating cobalt and nickel are the most used compositions for anodic and cathodic electrodes, respectively [10]. The main problem of these systems relates to the high sensitivity of the KOH electrolyte to ambient CO_2_ and the subsequent production of K_2_CO_3_. The resulting decrease in the number of hydroxyl ions lowers the ionic conductivity, while the precipitation of K_2_CO_3_ produces the clogging of the pores of the anode gas diffusion layer and a reduction of the ion transfer through the diaphragm [10,12,13], both effects jeopardizing the hydrogen yield production.

#### 1.1.2. Proton Exchange Membrane Water Electrolysis (PEMWE)

In the proton exchange membrane water electrolysis cells, proton conductor polymeric membranes (perfluorosulfonic acid membranes), acting as a solid electrolyte, are used to separate the anode and the cathode [6]. Cells run at low temperatures (25–80 °C) [6] and use titanium bipolar plates that are compatible with the existing corrosive operation conditions [8].

PEMWE provides interesting advantages vs. AWE. For example, it renders high current densities, a fast response under intermittent electrical energy supply, and a compact system design enabled by the absence of liquid electrolyte [14,15]. Additionally, the gases’ cross-over problem through diaphragms in alkaline electrolysis is significantly reduced in PEMWE [9]. Additionally, this technology offers the possibility of using a high-pressure H_2_ stream in the cathode compartment, while operating at atmospheric pressure in the anode chamber [6,9,10]. However, a high cost is a clear drawback of this type of cell. Firstly, it uses expensive noble metals such as Ir, Ru of Pt as a catalyst for the anode and the cathode, a requirement imposed by the need for a high electrochemical stability in the corrosive acidic medium where they have to operate. A related limitation is the high polarization voltage that has to be applied to the anode (~2.0 V) to achieve an efficient working regime [6,16]. A high cost is also a concern that affects the solid electrolyte membrane. For this purpose, Nafion^TM^ proton exchange ionic membranes are commonly used. These membranes comply with the requirements of a great ionic H^+^ conductivity and an extraordinary high chemical, thermal, and mechanical stability [17], though at the expense of a high cost. 

#### 1.1.3. Anion Exchange Membrane Water Electrolysis

Anion exchange membrane water electrolysis is a relative new technology that aims at combining the advantages of AWE and PEMWE, overcoming some of their limitations [18]. This technology has been scarcely investigated so far (the words “anion exchange membrane water electrolysis” typed in SCOPUS render fewer than 62 references, with ~75% of them published since 2019). 

The AEMWE cells work in weak alkaline media, typically in low concentrations of KOH, or other alkaline solutions such as 1% K_2_CO_3_ or even distilled water [19], thus under a less corrosive environment than AWE. However, a common working paradigm in this topic is the utilization of working conditions with a large amount of KOH. This means that more in-depth studies are required to maximize cell performance with low KOH concentrations in aqueous solution. Weak alkaline operation conditions of the AEMWE cells are compatible with cheap electrode materials, mostly based on Ni and Co, that can be similar to those used in traditional AWE. They also enable the use of membranes cheaper than those incorporated in PEMWE cells [8,19,20]. These anion exchange membrane (AEM) are polymeric membranes that replace the traditional AWE diaphragm circumventing the gas cross-over between the anodic and cathodic chambers [6]. In addition, the use of distilled water or low concentrated alkaline solutions significantly reduces the problems associated with the K_2_CO_3_ formation, already mentioned as a crucial limitation by the traditional AWE cell configurations [10,19,21,22]. Another advantage associated with the AEMWE technology is the compact character of the cells and the low temperatures (25–70 °C) of operation [23]. For all these reasons, AEMWE technology has emerged as a promising alternative, complying with the conditions of a low cost of the integrated components and a straightforward operation. The numerous advantages mentioned have led other authors to focus their attention on this technology in recent years. Nevertheless, these studies have mainly focused on comparing the critical anion exchange membrane cell components and efficiencies [6,10,11,24,25]. On the other hand, in this review, a particular attention is paid to the catalyst preparation methods since they are critical for the yield optimization and the setting of optimum working conditions for these cells, which has not been previously studied. The principles of operation and a detailed description of the state of the art about the characteristics and manufacturing procedures of the components of the AEM-type electrolysis cell will be also addressed in the following sections.

## 2. Basis of AEMWE Cell and Electrochemical Reactions

**Figure 3:**
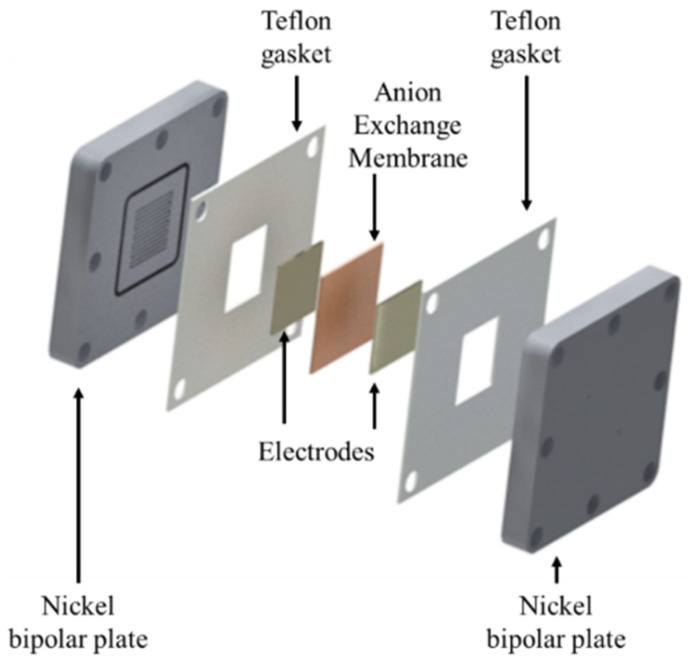
Schematic view of an AEMWE cell assembly and their components.

Figure 3 shows a schematic view of a conventional symmetric AEMWE cell. It consists of two bipolar flow field plates, two Teflon gaskets, and the membrane electrode assembly (MEA). The bipolar plates should be corrosion-resistant and have high electrical conductivity. Typical materials for the bipolar plates immersed in the cell alkaline environment are titanium [26,27], nickel [28,29], or graphite [30,31], although nickel is particularly recommended [32]. A fluidic circuit is engraved in the plates to guarantee an even liquid electrolyte flow through the electrodes. Besides their closing function, the Teflon gaskets are used for electrical isolation between the two bipolar plates. The components of the MEA are described in detail in Section 3.

In the AEMWE, hydrogen and oxygen are produced from water using an external power supply. The overall process consists of two half-cell reactions, the hydrogen evolution reaction (HER) and the oxygen evolution reaction (OER), that take place at the cathode and the anode, respectively. They can be described as [10]:

(1) $$\mathrm{HER}\;\left(\mathrm{cathode}\right){:\;\;\;\;\;4H}_{2}{O+4e}^{-}\to {2H}_{2}{+4\mathrm{OH}}^{-}{\;\;\;\;\;\;E}_{0}=-0.828\;V$$

(2) $$\;\;\;\;\;\mathrm{OER}\;\left(\mathrm{anode}\right){:\;\;\;\;\;4\mathrm{OH}}^{-}\to {\;O}_{2}{+2H}_{2}{O+4e}^{-}{\;\;\;\;E}_{0}=+0.401\;V$$

(3) $${\mathrm{Overall}\;\mathrm{reaction}:\;\;\;\;\;2H}_{2}O\;\to {2H}_{2}{+O}_{2}{\;\;\;\;\;\;\;E}_{0}=+1.230\;V$$

Water is fed to the cathodic compartment where it becomes reduced with electrons to produce hydrogen and OH^−^ ions (HER) (1). The OH^−^ ions pass through the membrane to the anode, where they become oxidized, producing oxygen and water (OER) (2). The overall process is the splitting of water molecules into hydrogen and oxygen molecules, as detailed in the overall reaction (3).

## 3. Materials in the Membrane Electrode Assembly 

The membrane electrode assembly is the active element in the AEMWE. It is formed by an anion exchange membrane sandwiched between the anode and cathode electrodes. The electrodes mainly consist of specific catalyst/ionomer coated macroporous gas diffusion layers. 

### 3.1. Anion Exchange Membranes

The anion exchange membrane (AEM) is a key component of the cell [10,33]. Its main function is the transport of hydroxyl ions from cathode to anode. It also acts as a barrier for the gases and other subproducts of the electrocatalytic reactions [6]. It consists of a polymer backbone functionalized with anion exchange groups, such as quaternary ammonium salts [34,35]. Their main requirements are a high ionic conductivity [22], a high mechanical and thermal stability, gas tightness [36,37], and stable long-lasting operation [6]. Additionally, a low cost is an implicit condition to bear in mind for practical applications.

It has been reported that the membrane conductivity can be improved by the nucleophilic substitution of the ammonium groups or by the substitution of methyl groups by hydroxyl ions [10]. Although further increases in the ionic conductivity can be achieved by a massive incorporation of anion exchange groups, this may reduce the mechanical strength due to an increase in water uptake and associated swelling [10]. 

The most commonly used commercial membranes in AEMWE are Fumasep^®^ FAA-3, Sustainion^®^ 37-50, Tokuyama A201, Aemion^TM^, and Orion^TM1^ membranes [36]. Their main characteristics are detailed in Table 1. They are supplied in their bromide (Br^−^) or chloride (Cl^−^) forms, so they have to be pre-treated to replace these ions by hydroxide (OH^−^) groups before their incorporation in AEMWE cells. This is generally achieved by immersion in NaOH or KOH solutions for, at least, 24–48 h [38,39,40,41].

Some studies have assessed the performance and stability of these membranes [37,43]. Liu et al. [37] demonstrated that the Sustainion^®^ 37-50 membrane had a lower resistance than Fumasep^®^ FAS-50, Fumasep^®^ FAPQ, AMI 7001, Nafion 115, or Celazole PBI using electrochemical impedance measurements. These authors obtained a current density of about 3.5 A cm^−2^ at 60 °C with a 1.0 M KOH electrolyte solution, using NiFe_2_O_4_ as the anode and NiFeCo as the cathode catalysts with loadings of 2 mg cm^−2^. They also proved the long-term stability of the system, applying a current density of 1.0 A cm^−2^ at 60 °C over more than 1950 h and obtained a low degradation rate of 5 µV h^−1^. Pushkareva et al. [43] also demonstrated that the Sustainion^®^ membrane had a better performance than Aemion^TM^ and Tokuyama A201 membranes at different electrolyte and temperature operation conditions, obtaining density currents higher than 3.0 A cm^−2^ at 60 °C and 1.0 M KOH as electrolyte. 

Promising long-term stability in the performance of these membranes has also been reported in the last years. Thus, only small degradation rates of 5 μV h^−1^ have been reported for the aforementioned Sustainion^®^ membranes for 2000 h operation at a fixed current of 1.0 A cm^−2^ [37], of 150 μV h^−1^ for a Tokuyama A201 membrane in 1000 h test maintaining a constant current of 470 mA cm^−2^ [44], or 800 μV h^−1^ for a Fumasep^®^ FAA-3-50 in a 1000 h operation test [45]. These values approach the low degradation rates associated with the PEM-type electrolyzers (for example, a decay of 6.8 μV h^−1^ was obtained at a fixed current of 2.0 A cm^−2^ in a 5000 h test [46]).

### 3.2. Gas Diffusion Layers

The AEMWE electrodes mainly consist of an active catalyst phase deposited or distributed on a macroporous support or gas diffusion layer (GDL). The catalyst can be coated onto the substrate (catalyst-coated substrate, CCS) or directly onto the membrane (catalyst-coated membrane, CCM). The role of the GDL, besides supporting the catalyst phase, is to allow electronic conductivity between the catalyst sites and the bipolar plates, to polarize the catalyst loads to activate the OER and HER reactions, and to provide a removal path for the gaseous products [6] of the hydrogen and oxygen evolution reactions. 

GDL supports made of carbon paper or cloths [30,47,48], Ti papers [41,49], stainless steel (SS) felt [37,50,51] or Ni foam [52,53] have been used for the anode. Ni or Ti metals show a high thermodynamic stability when acting as anode GDL in an alkaline medium. SS substrates generally passivize at anodic potentials in an alkaline environment, thus ensuring their stability [6]. On the other hand, long-term use of carbon anode GDL may have some limitations due to stability problems associated with the fact that OH^−^ ions are excellent nucleophilic intermediates and accelerate the carbon degradation [54]. On the cathode side, carbon paper [48,53,55], Ni [56,57], or SS [52] foams are often used as electrode supports.

### 3.3. Catalyst Materials

Currently, the most widely used catalysts used for AEMWE are transition metals such as Co, Ni, or Fe. This choice is supported by their electrochemical stability, low cost, and easy accessibility [58,59]. In particular, non-PGM (non-platinum group metal) commercial catalysts Acta 3030 (CuCoO_x_) and Acta 4030 (Ni/CeO_2_-La_2_O_3_/C), for OER and HER, respectively, are extensively used [10]. Their incorporation in the cells release the most common limitation of PEM electrolyzers, i.e., the high cost of the Pt- or IrO_2_-based catalysts [16]. Below, we perform a survey of the AEMWE catalysts most recently reported in the literature. However, although many authors are currently developing suitable electrodes for AEMWE, these have been rarely scaled up to fit complete cells.

#### 3.3.1. OER Catalysts

The OER is the limiting step of the complete electrolysis process due to its lower kinetics and higher overpotential than the HER [60,61]. For this reason, most studies related to AEMWE catalysts focus on the optimization of the OER catalyst. Among the most promising non-noble catalysts, Ni and Ni-alloys, Co mixed oxides, or graphene have demonstrated a high stability and activity for the OER [10]. Thus, in recent studies, it has been demonstrated that the addition of Fe to Ni catalysts [62] decreases the overpotential for the OER and increases the overall reaction performance [62]. The incorporation of Cu in the Co_3_O_4_ spinel structure has demonstrated the same effect, decreasing the onset potential of the OER [63]. Similar results have been also reported for other Co-based catalysts incorporating Li, Ni, or Cu [64]. 

**Figure 4:**
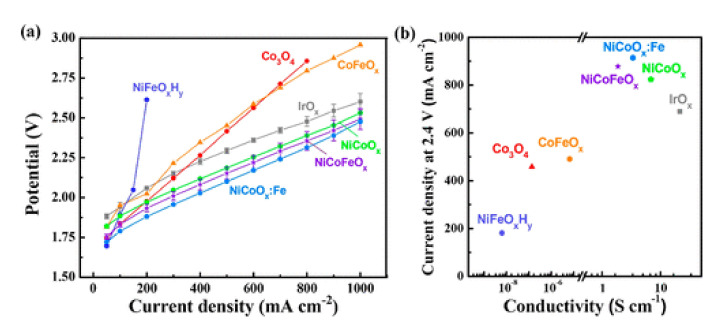
(**a**) Initial polarization curves of AEM water electrolyzers with several OER catalysts. (**b**) Current density in the electrolyzer at fixed applied voltage (2.4 V) correlated with the corresponding catalyst electrical conductivity. Reprinted with permission from [41]. Copyright 2019 American Chemical Society.

An interesting work dealing with OER catalysts has been published by Xu et al. [41]. These authors systematically tested a series of first-row transition-metal (oxy)hydroxide powders as anodic catalysts for AEMWE, following the idea that, under OER conditions, the active phases at the catalyst surfaces are the (oxy)hydroxides of the said transition metals [6]. For these electrochemical tests, a platinum loading of 3.0 mg cm^−2^ was used as the cathodic electrode mixed with 15 wt.% of FAA-3 ionomer in the catalyst ink. This was sprayed onto carbon paper (for the cathode) and titanium frit (for the anode) gas diffusion layers [41]. From the set of studied anode formulations, NiCoO_x_:Fe electrodes presented the best performance for AEMWE, reaching a current density of about 900 mA cm^−2^ at 2.4 V (see Figure 4a). These authors also found that the highest performance for Ni-Co oxide catalysts correlates to their higher electrical conductivity in comparison with other anodes (see Figure 4b). 

#### 3.3.2. HER Catalysts

Currently, CuCoO_x_, Ni-alloys, and graphene are extensively used as HER catalysts [10,65,66,67]. Among them, Ni can be considered the most promising catalyst for AEMWE since it can be used both as anodic and as cathodic catalysts for this type of process. Some authors such as Kim J. et al. [68] have investigated the HER activity improvement of nickel after adding a series of transition metals such as Fe, Cr, and Ti. They found that the Cr-Ni catalyst shows the highest HER performance in a 1.0 M KOH solution. 

Some works have also addressed the need of reducing the cost of the whole electrolyzer, following the effect of the amount of catalyst at the cathode while varying other process variables in order to maintain the system performance. For example, Pavel, C.C. et al. [44] varied the catalyst load on the cathode between 0.6 and 7.4 mg cm^−2^, fixing the amount of anode catalyst at 36 mg cm^−2^ and obtaining higher current densities when the amount of Ni/(CeO_2_-La_2_O_3_)/C cathodic catalyst reached the maximum of 7.4 mg cm^−2^. Lopez-Fernandez, E. et al. [47] also studied the influence of the amount of catalyst both in the half-cell and in a complete AEMWE cell, obtaining an optimum behavior for 0.38 mg cm^−2^ of Ni cathode catalyst loading.

### 3.4. Ionomers

Ionomers are polymeric organic molecules that act as a binder of the catalyst particles to the GLD support and the AEM, creating additional ion transport pathways between the reaction sites at the catalyst and the ionic exchange membrane [10,69,70]. This results in an increase in the electrocatalytic activity of the cell. Most MEA preparation methods use ionomers either applied directly on the GLD or AEM surfaces or incorporated into the catalytic inks used to prepare the electrodes [19,23,45,48]. 

For AEMWE cells, there is no standard ionomer, as in the case of Nafion^®^ for PEMWE [71,72,73,74]. Polysulfone (PSF) is a very popular one because it presents high thermal and chemical stability and low cost [75,76]. Other traditional ionomers for AEMWE are quaternary ammonia polysulfone (XQAPS) [77], Fumion FAA-3, from FumaTech company [41], Aemion^TM^ supplied by Ionomr Innovations Inc. [78], I_2_ from Acta Spa [19], or AS-4 from Tokuyama Corporation [76]. The proportion of ionomer and catalyst load at the electrodes is often optimized to obtain the best electrocatalytic performance of the electrolyzer.

In a general study on AEMWE, Vincent, I. et al. [19] studied the effect of the amount of ionomer (I_2_ ionomer, from Acta Spa) added to the catalyst ink on the electrochemical cell performance. They varied the ionomer load between 9–33% of the total catalyst ink and found that, within this variation range, the best performance was achieved with the lowest load. They also found that lower ionomer loads than 9% resulted in the formation of some cracks in the catalyst layer, while ionomer amounts of 25% and 33% resulted in a significant voltage drop. Using the optimal ionomer content, 30 mg cm^−2^ CuCoO_x_ (Acta 3030) anode, and 7.4 mg cm^−2^ Ni/(CeO_2_-La_2_O_3_/C) (Acta 4030) cathode commercial catalyst loads, these authors obtained a current density of 500 mA cm^−2^ at 1.9 V polarization voltage.

**Figure 5:**
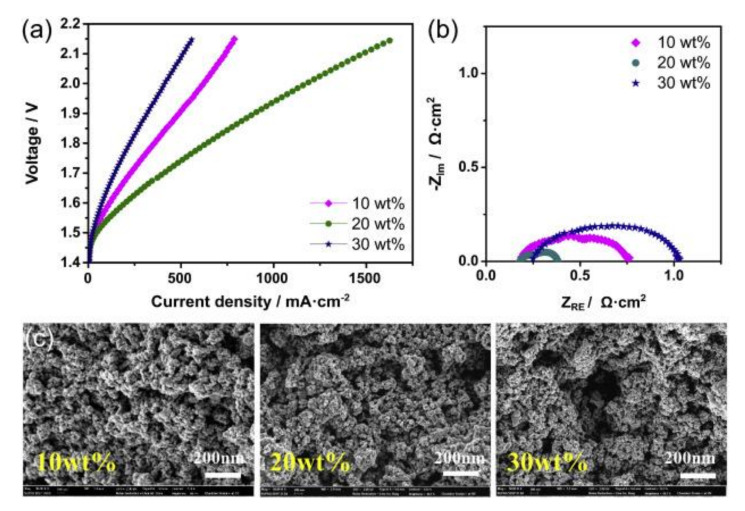
(**a**) Polarization curves and (**b**) Nyquist plots acquired at 50 °C for AEMWEs with several ionomer contents (10, 20, and 30 wt%). (**c**) FE-SEM images of MEAs fabricated using different ionomer contents. The feed condition was 1.0 KOH solution in both electrodes with a flow rate of 1 mL min^−1^. Reprinted from [23] with permission from Elsevier.

In another study by Park, J.E. et al. [23] on the optimization of the FAA-3-Br ionomer content in a MEA composed by IrO_2_ as the anodic catalyst, Pt/C as the cathodic catalyst, and Fumatech FAA-3-50 as the anion exchange membrane, these authors obtained the best MEA performance with a 20 wt% ionomer content (see Figure 5). This proportion is optimal to obtain a good trade-off between the number of active sites available to promote the electrochemical reaction and the appropriate pore morphology for an optimum diffusion of reactants and products. Figure 5 reveals that cell performance increases and the charge-transfer resistance decreases when the ionomer content increases from 10 to 20 wt%. This trend is attributed to an improvement of the reactant transport up to the active sites of the catalyst thanks to an increase in the size of secondary pores developed in the catalyst layer. This effect can be observed in the SEM images reproduced in Figure 5c. However, this tendency broke for higher ionomer loads, when the charge transfer, the mass transport, and ohmic resistances increased. This deleterious effect of high ionomer loads is justified by the coverage of the active sites by the ionomer molecules and the detachment of some catalyst particles that would block the pathway to the reactants. Using this ionomer/catalyst proportion and optimizing other operation parameters such as the OER catalyst load to 4 mg cm^−2^, these authors reached current density values of approximately 2.0 A cm^−2^ at 2.1 V in 1.0 M KOH electrolyte [23]. 

## 4. Preparation Methods for Catalyst Coated GDL for AEMWE

Due to the special characteristics and constrains of the electrolyzer cells, it is not only necessary to make a good choice of catalyst composition but also to optimize the integration structure of the catalyst within the electrode, the MEA, and the complete cell. This makes it important not only to choose the best formulation of the material catalyst for the specific anode and cathode reaction or to use the most appropriate ionomer type and load but also to select the best processing procedure for the integration of the catalyst material within the cell. 

The most common methods to fabricate catalysts for low-temperature water electrolyzers consist of traditional wet routes: co-precipitation, hydrothermal methods, or sol–gel. Common steps involved the preparation of the precursor solutions, the precipitation process itself, washings, filtrations, drying (temperatures vary between 50 and 120 °C), or calcination. In addition, in some cases, a preliminary milling step is necessary to produce an initial fine catalyst powder. 

Once the catalyst powder has been prepared, a catalyst ink/slurry is prepared that, in a second step, is sprayed or painted onto the GDL support [20,79]. A drawback of these wet routes is the use of solvents and the release of unwanted wastes that are potentially dangerous for the environment [80]. Another drawback is the large number of steps, which hinders scaling up the procedure and limits the reproducibility of the catalyst reactivity. 

In the last years, alternative catalyst-coated GDL electrode fabrication methods have been used to solve the aforementioned problems associated with the traditional wet fabrication routes. These include electrodeposition processes and other dry methods of film deposition such as chemical vapor deposition (CVD), atomic layer deposition (ALD), ion beam sputtering deposition (IBSD), or magnetron sputtering (MS). Below, we present a brief survey of the most recent preparation methods of catalysts for AEMWE cells, together with the description of the performance achieved in some representative examples from the literature.

### 4.1. Wet Routes

#### 4.1.1. Co-Precipitation Method

One of the most widely used methods to fabricate catalysts for AEMWE is the co-precipitation technique. By this method, salts of the active metal are dissolved and mixed to induce the nucleation and growth of a solid precursor incorporating the active metal phase. After precipitation, some washing steps are applied to remove residual components that may cause particle sintering and undesired agglomerations. Then, after filtering and drying, a powder is obtained. This powder is often annealed or calcined to obtain the desired crystalline catalyst phase and eventually grinded. Following this procedure, large catalyst amounts can be prepared where the catalyst particles are small [81]. The final catalyst powder is used to prepare a catalyst slurry or ink that is deposited onto the macroporous gas diffusion layer supports, usually by ink spraying or ink painting. 

As an example of this procedure, Li, H. et al. [59] fabricated, by co-precipitation followed by calcination under an argon atmosphere at 400 °C, Co_2-x_Ni_x_O_2_ (0 < x < 1.0) nanostructures with different Co/Ni molar ratios. The catalyst had a large specific surface area of 60.63 m^2^ g^−1^ and for a catalyst load of 0.1 mg cm^−2^. They reach current densities of 125 mA cm^−2^ at 2.0 V for the overall water-splitting without membrane in a 1.0 M KOH solution. Hao, G. et al. [82] obtained nanostructured mesoporous NiCo_2_O_4_, Co_3_O_4_, and NiO spinel oxides using a modified co-precipitation method based on the addition of ethylene glycol at room temperature. The precipitate was separated by centrifugation and dried in air, either at 80 °C for 24 h and at room temperature for 8 h. The thus-fabricated electrodes had 2 mg cm^−2^ of catalyst loading. Through a systematic comparison of Tafel slopes, current densities (at constant potential 600 mV vs. Ag/AgCl), and overpotentials (at constant current densities of 100 mA cm^−2^), an optimum Tafel slope of 68 mV dec^−1^ and overpotential of 717 mV at 100 mA cm^−2^ were obtained for a NiCo_2_O_4_/Ti electrode prepared at 350 °C. As another example, Liu, M. et al. [83] synthesized, through a co-precipitation method at room temperature, cobalt–iron pyrophosphate porous nanosheets with 1 mg cm^−2^ of catalyst loading to be used as OER electrodes. The catalyst was dried at 80 °C overnight. They obtained current densities of 135 mA cm^−2^ at about 1.58 V vs. RHE (iR corrected values). 

#### 4.1.2. Hydrothermal Method

Hydrothermal method is another useful technique to obtain nanostructured catalysts. Crystal growth is carried out in an equipment consisting of a steel pressure vessel acting as an autoclave [84]. This technique involves the use of a solvent and various precursors and provides an efficient way to control the size and structure of the synthetized nanoparticles [85]. The main advantage is the application of low temperatures under conditions enabling the direct crystallization of the oxides and avoiding annealing steps at elevated temperatures.

For example, using a modified hydrothermal method, Xu. D, et al. [41] synthesized a wide range of metal oxide catalysts dissolving a metal acetate hydrate precursor in a mixture of ethanol, water, and ammonia. This solution was stirred for 15 min, and the resulting suspension was heated for 3 h at 150 °C. The metal oxides nanoparticles were centrifuged and washed several times with ethanol and then dried at 80 °C overnight. Then, the catalyst powder was suspended in isopropanol and sprayed onto the gas diffusion layers until reaching a loading of 3.0 mg cm^−2^. After spraying the ink, an ionomer solution was sprayed on the top of the catalyst layer. The authors obtained 370 mA cm^−2^ of current density, with their NiCoO_x_Fe optimum anode catalyst and Pt catalyst as cathode, at 2.0 V and 1.0 M KOH solution. 

#### 4.1.3. Sol–Gel Method

Due to their versatility, sol–gel methods are widely used to prepare catalysts with different compositions, homogeneity, and structure [86]. Sol–gel catalysts are formed through kinetically controlled reactions starting from molecular precursors of the components integrated in the final materials catalyst [87]. Maruthapandian, V. et al. [88] studied the influence of Ni and Co added to spinel ferrites (MFe_2_O_4_) fabricated by sol–gel. The results demonstrated that the bare NiFe_2_O_4_, without Co, presented the best electrochemical activity for OER in an alkaline medium (about 68 mA cm^−2^ of current density at 2.0 V vs. RHE). The prepared NiFe_2_O_4_ catalyst powders were prepared by annealing at 700 °C for 3 h. Then, the catalyst ink was prepared by mixing the powder with acetylene black carbon and polyvinylidene fluoride (PVDF) as a binder in N-methyl-2-pyrrolidone (NMP), and the resulting slurry was then coated on the carbon paper substrate by the doctor blade method until reaching around 2 mg cm^−2^ of catalyst load. Other groups have synthesized nanoparticles of Co-Cu alloys by sol–gel using cobalt acetate and copper acetate as precursor and poly(vinyl alcohol) as solvent. A calcination step in a controlled environment (900 °C for 7 h in an inert medium with reducing gases, H_2_ and CO) is necessary to induce the complete reduction of the acetates to pure metals. The catalyst ink was drop-casted onto a glassy carbon and the optimum composition electrode, Co_0.95_Cu_0.05_, was demonstrated to have a good performance for both OER and HER in alkaline medium, as well as a high stability after 1000 cycles. They obtained about 180 mA cm^−2^ for the overall water splitting reaction (without membrane) at 2.0 V and 1.0 M KOH solution [89]. 

### 4.2. Thin Film Deposition Routes

#### 4.2.1. Electrodeposition

Electrodeposition is one of the most widely used fabrication technique of catalysts for water electrolysis. It is an electrochemical technique that combines electric-charge-induced diffusion and chemical transformation using redox reactions at the anode and cathode [90]. The deposition of the catalyst on the desired substrate (in this case, on a suitable GDL) is carried out, applying a direct current to the electrodes immersed in an electrolyte solution. This electrolyte solution contains cations of the metal that, under the applied electrical field, move to the electrode and are deposited in metal form [91]. Using this technique, the metal catalyst becomes deposited only at conductive sites of the electrode support [92]. Adjusting process parameters (composition of the electrolyte, applied voltage, geometrical aspects within the electrodeposition cell, temperature, etc.), this procedure renders films with different nanostructures and compositions. It also has the capacity to fabricate one-dimensional nanostructures such as nanorods, nanowires, nanotubes, nanosheets, flower-like nanostructures, etc. [90]. Electrodeposition is a fast and low-cost process [93] that, combined with other synthesis procedures, has been applied to prepare high-performance and outstanding catalysts [94,95,96]. 

**Figure 6:**
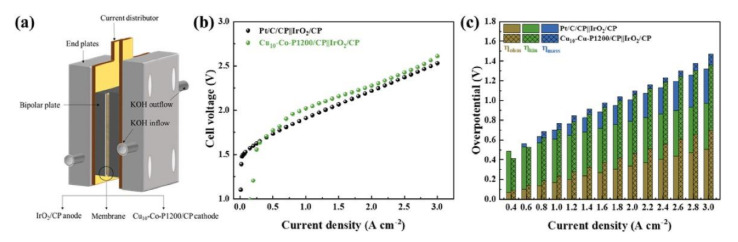
(**a**) Schematic illustration of AEMWE single-cell configuration. (**b**) Polarization curves of AEMWE single cells with IrO2/CP anode coupled with a commercial Pt/C/CP or Cu10–Co–P1200/CP cathode. (**c**) Overpotential subdivisions of (**b**). Reprinted from [66] with permission from Elsevier.

There are many examples in the literature where electrodes for AEMWE have been synthesized using this procedure. For example, Ren, H. et al. [97] synthesized Co_0.9_Fe_0.1_-Se/NF electrodes using one-step electrodeposition in alkaline media, both for OER and HER processes. They deposited about 30 mg cm^−2^ of catalyst loading into a nickel foam substrate with a great stability and performance after a 36 h test. They also demonstrated that Fe incorporation may significantly promote the OER process through the lowering of the overpotentials to values of 246 and 125 mV at 10 mA cm^−2^ for OER and HER, respectively. Zhang, A. et al. [98] fabricated electrodes by this technique in a three-electrode electrochemical system at room temperature containing phosphor on carbon paper GDL with a low amount of catalyst (about 0.11 mg cm^−2^) and Co-Ni-P, Co-P, or Ni-P composition. These electrodes could be used as both anodes and cathodes and depicted a high performance and stability after 10 h of testing. The Co-Ni-P catalyst presented the best electrochemical performance, obtaining approximately 68 mA cm^−2^ at 1.9 V. Guo, W. et al. [66] fabricated Cu-Co-P catalysts by electrodeposition on carbon paper, varying the catalyst loading and the Cu content. The electrode exhibiting the highest performance was used as the cathodic electrode, while a commercial IrO_2_ was used as the anode in an AEMWE. The highest electrochemical performance for water electrolysis was found for a catalyst mass loading of 3.13 mg cm^−2^, obtaining 1.4 A cm^−2^ of current density at 2.0 V in 1.0 M KOH solution. Figure 6b shows that the Cu-Co-P electrode rendered current densities values slightly lower than those obtained with a Pt electrode, but the authors confirmed that the performance was superior to that reported for non-noble metals. Moreover, they found (Figure 6c) that, although the Cu-Co-P cell presented a larger ohmic overpotential (η_ohm_) and kinetic overpotential (η_kin_), its low mass transfer overpotential (η_mass_) resulted in a highly performant cell, with an activity close to that of a Pt electrode cell. 

Aiming at reducing the amount of catalyst and maximizing the electrocatalytic performance, Han, S. et al. [99] deposited by electrodeposition different loadings of a Co-Doped Fe_3_O_4_ thin film catalysts and found that an optimum thickness of 550 nm rendered 100 mA cm^−2^ at approximately 1.83 V vs. RHE for the OER. Meanwhile, Pei, Y. et al. [100] obtained a maximum activity using a Co-Ni-P electrodeposited thin film with a thickness of 1.63 µm. They reached 100 mA cm^−2^ at approximately 1.8 V for the overall water electrolysis (without membrane) in 1.0 M KOH. 

#### 4.2.2. Chemical Vapor Deposition

CVD is a dry method where a catalyst thin film becomes deposited on the substrate by its exposure to one or more volatile precursors [101]. In CVD, a thermally induced chemical reaction takes place between a mixture of precursor gases on the surface of the substrate material. The chemical decomposition and/or reaction of specific gaseous precursors gives rise to a solid coating layer [102]. CVD is a very versatile deposition technique that allows the synthesis of monocrystalline, polycrystalline, and amorphous phases [102]. 

CVD presents some disadvantages, such as the requirement of an additional step for the fabrication of the catalyst-coated GDL or the fact that it does not permit an easy control over the film stoichiometry. In addition, CVD generally requires high temperatures at which some nanostructures may be unstable [101,103,104,105]. It is also noteworthy that the fabrication of some transition metals containing coatings via CVD may involve the use of precursors whose decomposition generates toxic or hazardous substances [80].

Chang, J. et al. [106] reported a mixed synthesis procedure using a solvent–thermal reaction followed by CVD that led to the deposition of Fe_2_P_2_S_6_ catalysts on carbon paper electrodes. They were used as the anode and cathode for half-reactions in a three-electrode electrochemical cell and in a complete AEMWE cell. With just 2 mg cm^−2^ of catalyst load, these authors obtained a current density of 580 mA cm^−2^ at 2.0 V in 1.0 M KOH solution and demonstrated a better stability in a 24 h test than when using a Pt-IrO_2_ catalyst. Kuang, E. et al. [107] fabricated bimetal (Ni/Mo) sulfide-based catalysts from NiS_2_ and MoS_2_ nanoparticles using a hydrothermal method followed by an in situ CVD treatment. These electrocatalysts were highly efficient for HER, presented low overpotentials, and enabled high current densities in acidic, alkaline, and neutral electrolytes. 

#### 4.2.3. Atomic Layer Deposition

ALD is a variant of the CVD where gas precursors are introduced in a reaction chamber to form a thin film through chemical surface reactions. In ALD, the precursors are sequentially pulsed into a deposition chamber to avoid reactions in the gas phase. The successive self-terminated surface reactions of the reagents cause the growth of the desired material composition with an excellent conformity and uniformity of film thickness [108].

The use of this technique has been reported several times for the fabrication of AEMWE electrode catalysts. Haschke, S. et al. [109] studied nanoporous SnO_2_/Fe_2_O_3_/IrO_2_ thin films fabricated in a three-step process. They deposited on an alumina substrate 20 nm of SnO_2_ followed by 10 nm of Fe_2_O_3_ and 10 nm of IrO_2_. Then, a 400 °C annealing step was applied for 12 h. Using this experimental procedure, extremely low iridium loadings of 7.5 μg cm^−2^ were spread in the form of a homogeneous thin layer on the electrode surface. Nardi, K.L. et al. [110] fabricated NiO films by ALD and incorporated Fe from the electrolyte to increase the OER activity. They demonstrated that, from 1 to 18 nm equivalent thickness, the amount of Fe deposited did not have a significant influence on the redox behavior. This result suggests that, due to the dense nature of the ALD NiO films, only the outermost surface layers were active for OER.

To conclude, the ALD method produces homogeneous and compact layers [110,111] where the availability of active sites at the surface, a requirement for an optimum electrochemical performance for water electrolysis, is small due to the lack of porosity. For practical applications, however, a point to bear in mind is that the ALD method is strongly dependent on the design of the deposition reactor [108], thus procuring an extra difficulty in terms of sample reproducibility and scalability. 

#### 4.2.4. Ion Beam Sputtering Deposition

IBSD is a thin film deposition technique that utilizes an external ion source to cover the substrates with the material sputtered from selected targets. This technique has been only used in a few AEMWE studies, probably because it gives rise to dense coatings that are less suitable for their use as electrodes for water electrolysis [112]. It has been successfully used by Grigoriev, S.A. et al. [113] to prepare platinum nanoparticles using a target of graphite with platinum inserts. Before the deposition step, an impregnation–reduction method was used to synthesize the initial platinum-based catalyst. The final catalyst with a load of 1.0 mg cm^−2^ and a 15 wt. % of Nafion ionomer was used as the cathode. As part of this work, the authors compared the electrochemical performance of the catalysts supported on a carbon GDL using a combined method of impregnation–reduction for the first metal and ion beam sputtering deposition for the second metal. Using this electrode, they obtained 1750 mA cm^−2^ of current density at 2.0 V in 10 wt. % NaOH solution at 90 °C. 

#### 4.2.5. Magnetron Sputtering Deposition

Magnetron sputtering (MS) is a physical vapor deposition method based on the bombardment of a target material with the noble gas ions formed in a plasma discharge. When the momentum of the incident ions in the plasma discharge is high enough, their interaction with the atoms at the surface of the target causes their sputtering and deposition onto a given nearby substrate. This technique operates at room temperature, is highly reproducible, and can be easily scaled up for large area manufacturing at the industrial level [114,115]. MS is an easy-to-use, safe technique, and due to its dry character, it does not generate wastes that are potentially detrimental for the environment [80,116]. In addition, MS provides a strict control of composition and load of the catalyst film on the electrode. Using this one-step preparation technique, it is possible to prepare a large variety of chemical compounds: single metals [117,118], metal alloys from a single-target with the desired composition [119] or by co-sputtering using more than one target [120], oxides by reactive magnetron sputtering adding oxygen to the plasma discharge [31], nitrides, using N_2_ as reactive gas [121], etc. Additionally, control over the deposited microstructure is also possible, varying the angle of the particle flux with respect to the surface normal, i.e., by means of oblique angle deposition (OAD) [114]. 

To maximize the performance for water splitting, electrodes must present a high porosity and a large electrochemical active surface area [122]. The OAD-MS technique permits the fabrication of highly porous electrodes consisting of nanocolumns separated by large voids [115,123]. This nanocolumnar microstructure stems from atomic shadowing effects taking place during the electrode growth [124,125,126].

Selected cases of MS and MS-OAD thin films used as electrodes will be described next to exemplify the possibilities of these techniques for water electrolysis applications. Lian, J. et al. [127] fabricated amorphous Fe-Co-P-C films supported on carbon paper GDL, varying the ratio of Fe by means of a magnetron co-sputtering technique, and studied the effect of the content of composition on the electrocatalytic performance during the OER in a 1.0 M KOH electrolyte. They obtained an overpotential of 310 mV to reach 10 mA cm^−2^ for a Fe_20_Co_60_P_13_C_7_ film catalyst. In addition, they studied the stability of the samples after 2000 cycles and only found a slightly decayed electrode performance during the OER that they attributed to the formation of oxygen bubbles at the catalyst–solution interface. Using magnetron co-sputtering from a single target, Delvaux, A. et al. [128] prepared electrodes with different Al/Ni atomic ratios and studied their performance in a 1.0 M KOH solution. They obtained an optimum performance at a potential value of 1.58 V vs. RHE (iR-corrected) to reach 10 mA cm^−2^ and a low Tafel slope of 46 mV dec^−1^ for the catalyst with 0.2 Al/Ni at. ratio. Slavcheva, E. et al. [129], by means of reactive magnetron sputtering, deposited IrO_2_ films for their use in PEM-type water electrolysis. Films with thicknesses varying between 250 and 1000 nm, corresponding to a catalyst load between 0.1 and 0.4 mg cm^−2^, were tested, obtaining a maximum current density of 0.3 A cm^−2^ (at 1.55 V vs. RHE) with a 0.2 mg cm^−2^ loaded anode. These results demonstrated that loading can be straightforwardly controlled with the magnetron sputtering technique. In the same line, Chen, G. et al. [130] deposited Ba_0.5_Sr_0.5_Co_0.8_Fe_0.2_O_3-x_ by MS, varying the catalyst load in electrodes intended for alkaline water splitting. They obtained 90 mA cm^−2^ at 1.6 V in a complete cell without a membrane and 1.0 M electrolyte solution.

**Figure 7:**
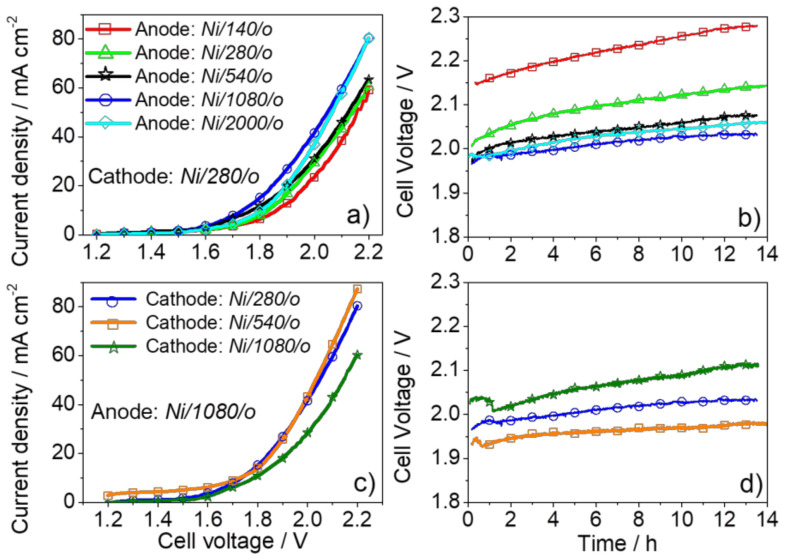
(**a**) LSV curves obtained for MEA assemblies integrating Ni/YY/o anodic electrodes of various equivalent thicknesses and a Fumapem membrane. (**b**) Constant-current (32 mA cm^−2^) chronopotentiometries measured for the assemblies in (**a**). (**c**) LSV obtained for MEA assemblies with Ni/YY/o cathodes of various equivalent thickness between 280 and 1080 nm and the optimum Ni/1080/o anode. (**d**) Constant-current (32 mA cm^−2^) chronopotentiometries measured for the assemblies in (**a**). All measurements were carried out in a 1.0 M KOH electrolyte solution at 40 °C. Reprinted with permission from [47]. Copyright 2020 American Chemical Society.

Meanwhile, using the MS-OAD technique, López-Fernández, E. et al. [47,55] found optimized current yields of 42 or 60 mA cm^−2^ at 2.0 V polarization and a 1.0 M KOH electrolyte solution with about 1 µm equivalent thickness of either Ni or Co_x_Cu_y_O_z_ anodic catalysts deposited on carbon paper GDL, respectively. Figure 7a shows the linear sweep voltammetries obtained for several nickel anodes and various amounts of catalysts for a fixed cathode load. Maximum current density values were obtained for the Ni catalyst with an equivalent thickness of approximately 1 µm. For this anode thickness, a minimum cell voltage was determined at a constant current (Figure 7b). The authors attributed this behavior to the nanocolumnar character of the nickel films deposited at a glancing angle and the fact that the available surface area will increase with the equivalent thickness of the catalyst films. The breakdown of this tendency for thicknesses above 1.0 µm was associated to particle agglomeration and degradation of the nanocolumnar microstructure. In the same way, studying the influence of the active phase load in the cathode (Figure 7c,d), an optimum cathode thickness of 540 nm was found. 

Unlike the catalysts fabricated with wet methods, the MS technique permits the preparation of catalyst films with different oxidation states and a similar microstructure and active phase loading [47]. For example, López-Fernández et al. [47] adjusted the chemical state of nickel-based electrodes, modifying the deposition conditions in a reactive MS process (i.e., changing the composition of the gas mixture in the magnetron plasma discharge). They obtained nickel electrodes in metallic, oxide, and oxyhydroxide chemical states, while keeping constant the amount of catalyst and their porosity. 

In summary, MS is a one-step method that could greatly simplify the manufacture procedures of electrodes [131,132]. Moreover, using MS in an oblique angle deposition configuration, the obtained catalyst-coated GDL electrodes show high specific electrocatalytic activity for water electrolysis. The high reproducibility and stability of the electrodes fabricated by this technique have also been demonstrated [47,55]. Other advantages of magnetron sputtering are the high adhesion of films to the substrates, a high deposition rate that permits a short manufacturing time for the electrodes, and the possibility to sputter any metal, alloy, or other components varying the gas plasma composition [133]. An additional advantage in terms of costs is the possibility to fabricate electrodes, either anode or cathode, at room temperature, decreasing the fabrication cost and time [47]. 

**Figure 8:**
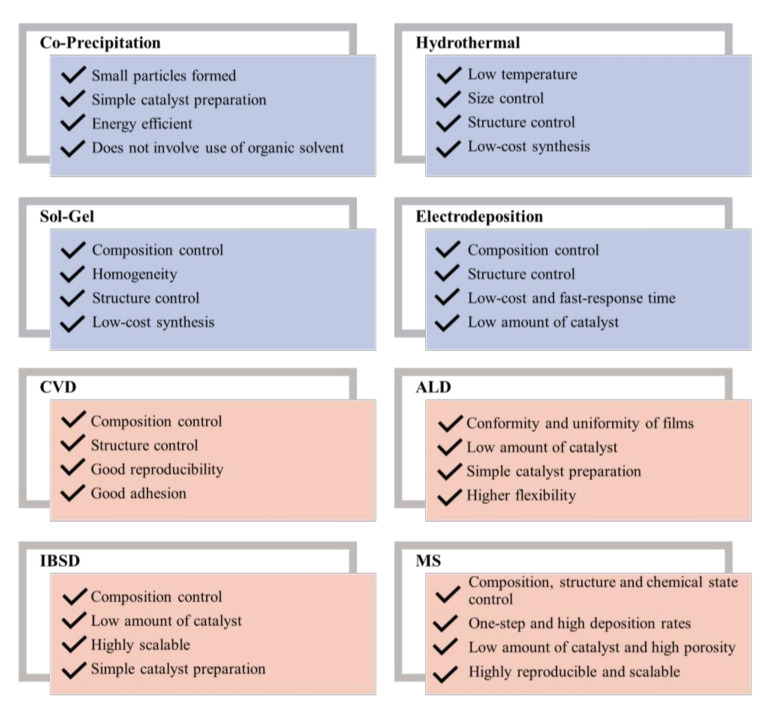
Summary of the main advantages of the catalyst deposition processes regarding their implementation in AEMWE.

Figure 8 shows a summary of advantages of various catalyst deposition processes intended for their implementation in AEMWE cells. 

## 5. AEMWE Cell Performance

Finally, to compare the AEMWE performance using different catalyst formulations and preparation methods, Table 2 gathers a series of examples from the literature including data such as the components of the MEA, the catalyst fabrication procedure, the operating conditions, and the current density obtained at a particular voltage. In addition, due to the importance of decreasing the catalyst cost, this table also includes a comparative assessment of the anodic specific activity. It is remarkable that although the absolute activity of AEMWE with catalyst electrodes prepared by MS is lower than that reported for other cells with similar catalyst composition, these electrodes are the most efficient in terms of specific activity. For example, specific activities well above 100 mA mg^−1^ [31,55] were obtained for cobalt–copper mixed oxides anodic catalysts deposited by magnetron sputtering, values that outperform those of other cells operated under similar conditions with catalysts of the same composition but prepared by other methods [19,41,134,135]. Otherwise, specific activities about 10 times higher were obtained for IrO_2_ electrodes prepared by electrodeposition, even using non-noble metals as a cathode electrode [66], compared to other wet methods such as the hydrothermal method [41].

## 6. Concluding Remarks and Future Prospects

In this review, we have provided a comprehensive overview of the basic operational principles and the state of the art of the components of AEMWE. Special attention has been paid to the methods utilized for the preparation of the electrode catalysts. The following specific points have been identified as requiring further improvements for the future deployment of this technology:

Many authors are developing suitable electrodes for AEMWE technology. Nevertheless, these electrodes have been rarely scaled up to fit a full electrolysis cell with an anion exchange membrane.

The chemical and mechanical stability of the membranes must be further improved in order to reach the stability values of the PEM electrolysis (today in the order of few μV h^−1^ degradation rate at 2.0 A cm^−2^ after several thousand hours of operation). 

The anion exchange membrane is compatible with the use water at neutral pHs or low concentrations of KOH, but more in-depth studies on this subject should be performed to maximize cell performance under these conditions. 

The most common methods to fabricate catalysts, such as co-precipitation, hydrothermal methods, or sol–gel, involve several steps in the process, such as washing, filtration, drying, or calcination. In addition, once the catalyst powder is manufactured, a catalyst ink must be prepared to be sprayed onto the GDL or membrane in a final stage.

Among the traditional catalyst manufacturing methods, electrodeposition has numerous advantages, such as the possibility of manufacturing catalysts with different compositions and nanostructures or being a fast and low-cost method. However, a drawback of this (and the other wet routes) method is the use of solvents and the release of unwanted wastes potentially dangerous for the environment.

To avoid dangerous wastes in liquid form, dry methods of film deposition such as CVD, ALD, IBSD, or MS have emerged in the last years as potential methods for the manufacture of catalysts. Among them, MS at oblique angle deposition appears as a suitable promising method of catalyst fabrication for anion exchange membrane water electrolysis. The procedure is simple, reproducible, and environmentally friendly and renders catalysts loads with a high porosity well-suited for AEMWE. 

The use of non-noble metal catalysts and cheaper membrane reduces the cell cost. Nevertheless, the overall performance should be increased to be competitive with the PEMWE and decrease the hydrogen production cost. 

## Figures and Tables

**Table 1:** Main characteristics of the most commonly used commercial AEMs.

Membrane	Company	Thickness/µm	Conductivity/mS cm^−1^	Ref.
Fumasep^®^ FAA-3	Fumatech	45–50	40–45	Technical data sheet
Sustainion^®^ 37-50	Dioxide Materials	50	70	[42]
Tokuyama A201	Tokuyama	28	42	[36]
Aemion^TM^	Ionomr	50	80	Technical data sheet
Orion^TM^	Orion Polymer	30	60	Technical data sheet

**Table 2:** A comparison of some of the most relevant studies of AEMWE.

Anode	Cathode	Membrane	Fabrication Method	Catalyst Loading (mg cm^−2^)		Current Density (mA cm^−2^)	Specific Activity * (mA mg^−1^)	T (°C)	Electrolyte	Potential (V)	Ref.
				Anode	Cathode						
NiCoO_x_Fe	Pt	FAA-3	Hydrothermal	3	3	370	123	50	1.0 M KOH	2.0	[41]
Co_3_O_4_	Pt	FAA-3	Hydrothermal	3	3	220	73	50	1.0 M KOH	2.0	[41]
IrO_x_	Pt	FAA-3	Hydrothermal	3	3	150	50	50	1.0 M KOH	2.0	[41]
Ni_90_Fe_10_/CeO_2_	Pt	FAA-3PE-30	Chemical Reduction	6	1	1930	322	50	1.0 M KOH	1.9	[136]
NiCo_2_O_4_	NiFe_2_O_4_	Self-preparation	Co-precipitation	2.5	2.5	165	66	45	15 wt% KOH	2.0	[137]
Ni	Ni	FAA-3-50	Magnetron Sputtering	0.78	0.38	42	54	40	1.0 M KOH	2.0	[47]
Ni	Ni	FAA-3-50	Magnetron Sputtering	0.38	0.17	31	82	40	1.0 M KOH	2.0	[47]
NiMn_2_O_4_	Pt	FAA-3-50	Oxalate	3	0.5	380	127	50	1.0 M KOH	2.0	[45]
IrO_2_	Pt	FAA-3-50	Commercial catalyst	4	0.4	1750	438	70	1.0 M KOH	2.0	[23]
Cu_x_Co_y_O_z_	Ni	FAA-3-50	Magnetron Sputtering	0.18	0.76	46	256	40	1.0 M KOH	2.0	[31]
Cu_x_Co_y_O_z_	Ni	FAA-3-50	Magnetron Sputtering	0.4	0.38	60	150	40	1.0 M KOH	2.0	[55]
Cu_0.72_Co_2.28_O_4_	Pt	Fumasep-30	Co-precipitation	10	1	1000	100	35	1.0 M KOH	1.9	[134]
CuCoO_x_	Ni/(CeO_2_-La_2_O_3_)/C	A-201	Commercial catalyst	30	7.4	650	22	60	1.0 M KOH	2.0	[19]
CuCo_2_O_4_	Pt	X-37-50 Grade T	Hydrothermal	23	1	1400	61	45	1.0 M KOH	1.9	[135]
IrO_2_	Cu-Co-P	Sustainion 37	Electrodeposition	3.13	-	1400	447	50	1.0 M KOH	2.0	[66]
Fe_2_P_2_S_6_	Fe_2_P_2_S_6_	YAB	Hydrothermal + CVD	2	2	580	290	50	1.0 M KOH	2.0	[106]
Ir	Pd/Pt	Nafion-115	IBS	1	1	1750	1750	90	10 wt% NaOH	2.0	[113]

* per amount of anodic catalyst.

## Data Availability

Not applicable.
